# Effect of Dietary Supplementation of Biological Curcumin Nanoparticles on Growth and Carcass Traits, Antioxidant Status, Immunity and Caecal Microbiota of Japanese Quails

**DOI:** 10.3390/ani10050754

**Published:** 2020-04-26

**Authors:** Fayiz M. Reda, Mohamed T. El-Saadony, Shaaban S. Elnesr, Mahmoud Alagawany, Vincenzo Tufarelli

**Affiliations:** 1 Poultry Department, Faculty of Agriculture, Zagazig University, 44511 Zagazig, Egypt; fayizreda@yahoo.com; 2 Department of Agricultural Microbiology, Faculty of Agriculture, Zagazig University, 44511 Zagazig, Egypt; m_tlatelsadony@yahoo.com; 3 Poultry Production Department, Faculty of Agriculture, Fayoum University, 63514 Fayoum, Egypt; ssn00@fayoum.edu.eg; 4 DETO—Section of Veterinary Science and Animal Production, University of Bari ‘Aldo Moro’, 70010 Valenzano, Italy

**Keywords:** biological nano-curcumin, growth, diet, immunity, antioxidant, pathogens, quail

## Abstract

**Simple Summary:**

Nanoparticles such as nano-curcumin are easier to pass through cell membranes in organisms and interact rapidly with biological systems. Therefore, using nano-curcumin is one of the recommendations for improving the bioavailability of curcumin, which would increase its absorption. Thus, this study focused on effects of nano-curcumin levels on the growth, carcass yield, blood chemistry and caecal microbiota of growing quails. From our results, supplemental nano-curcumin had beneficial impacts on growth, lipid profile, antioxidant, immunity of quail, and reduction in pathogenic bacteria.

**Abstract:**

This study was planned to evaluate the impact of different nano-curcumin levels on the growth rate, carcass, blood chemistry and caecal microbes of growing quail. A total of 270 Japanese quails at one-week-old were distributed to six equal groups; each group consisted of 45 unsexed birds with five replications (nine quails each). The 1st group was fed a basal diet, whereas the 2nd, 3rd, 4th, 5th and 6th groups were fed diets containing nano-curcumin (0.1, 0.2, 0.3, 0.4 and 0.5 g/kg diet, respectively). Nano-curcumin levels significantly increased (*p* ≤ 0.0001) body weight at 3 weeks and 5 weeks of age. Body weight gain during 1–3, 3–5 and 1–5 weeks of age was significantly increased (*p* < 0.0001) in groups treated with nano-curcumin levels (except at 0.3 g/kg; 1–3 weeks) compared to control. During 1 to 5 weeks, feed intake was decreased (*p* < 0.0001) in birds receiving nano-curcumin (0.1, 0.3 and 0.4 g/kg) diets. The best values of feed conversion ratio were recorded for the 0.4 g nano-curcumin-treated group. Carcass traits were not affected Nano-curcumin levels. The inclusion of nano-curcumin (0.2, 0.3 or 0.5 g/kg) significantly increased serum TP (*p* = 0.0004), albumin (*p* = 0.0078) and globulin (*p* < 0.0001). Quails fed with nano-curcumin (0.2 g/kg) exhibited the highest SOD and GSH activities, serum IgG and IgM concentrations and complement values compared to control. The addition of any level of nano-curcumin in the quail diet also significantly improved the lipid profile. In conclusion, supplemental nano-curcumin had beneficial impacts on growth, lipid profile, blood constituents, antioxidant indices, and immunity of growing quail, as well as increasing counts of lactic acid bacteria and reducing pathogenic bacteria.

## 1. Introduction

The general trend in the poultry industry is to provide a safer feed, to enhance physiological and productive indicators [1]. The effect of natural products on the capability of nutrients absorption in the gut is a major rationale for recent research. Several investigations have stated that plant derivatives included in poultry feeds deliver useful effects on performance, health, immune response and product quality [2,3,4,5]. One of these plant materials is curcumin. Curcumin is the principle active constituent of *Curcuma longa*. Curcumin has long been used in poultry feeds, owing to its favorable effects, including antimicrobial, antioxidant, anti-inflammatory, anti-inflammatory and immunostimulant properties [6,7]. Curcumin shows pharmacological efficacy and safety and contributes to the treatment of several diseases. It also improves the endogenous secretion of digestive enzymes [8] and reduces lipid peroxidation [9]. 

Curcumin can be used with nanotechnology that potentiates its useful effects. Nanoparticles are easier to pass through cell membranes in organisms and interact rapidly with biological systems [10]. Thus, using nano-curcumin is one of the recommendations for enhancing the bioavailability of curcumin, leading to an increase in its absorption [11]. It has been established that nano-curcumin displays improved bioavailability and distribution in the tissues [12]. The dietary supplementation of nanocurcumin displayed a significantly positive effect on performance [13]. Sayrafi et al. [14] clarified that the declined the liver enzyme activity following supplementation with nanocurcumin may be due to its antioxidant properties. Curcumin has an antioxidant function and adjusts the intestinal microbial composition [15]. Furthermore, Partovi et al. [16] stated that nanocurcumin at a level of 300 mg/kg diet can be used in poultry production as a good feed additive. However, the inclusion of nano-curcumin in quail diets during the growth period is still limited. 

Thus, the purpose of the current study was to determine the effects of different nano-curcumin levels on the growth, carcass yield, lipid profile, blood constituents, and antioxidant and immunological indices, as well as the caecal microbiota, of growing quails.

## 2. Materials and Methods

All experimental procedures of the study were performed according to the Local Experimental Animal Care Committee and approved by the ethics of the institutional committee of Department of Poultry, Faculty of Agriculture, Zagazig University, Zagazig, Egypt.

### 2.1. Source of Curcumin Nanoparticles 

Curcumin nanoparticles in this study were synthesized from *Bacillus subtilis* LA4, which was isolated from soil samples that were collected from different sites next to the plant rhizosphere in Sharqia Governorate, Egypt [17,18,19]. Under the optimum conditions of temperature, pH, incubation time, and other parameters, curcumin nanoparticles were produced. The characterization of the curcumin nanoparticles using modern devices and technologies was also performed to learn the properties of the curcumin nanoparticles obtained from the *Bacillus subtilis* LA4 bacteria. 

### 2.2. Biosynthesis of Curcumin Nanoparticles 

For the biofabrication of curcumin nanoparticles using the tested bacterium, 250 mL conical flasks containing 20 mL of supernatant from bacterial culture were separately mixed with 30 mL of 100 mg (0.27 mM) aqueous solutions of filtered sterilized curcumin, following the method of [20,21] with some modification. Then, the reaction mixture flasks were placed at 160 rpm in a shaker incubator at 30 °C for 72 h to allow the reduction process to occur. Furthermore, a set of flasks containing 20 mL of NB and 30 mL of 0.27 mM curcumin solution were prepared to confirm that the biotransformation of curcumin nanoparticles was only mediated by the use of bacterial cell-free extract [21].

### 2.3. Antibacterial Activity of Curcumin Nanoparticles

Fresh LB medium was used in all experiments to recover bacteria by sub-culturing. A tiny part from an inoculum of each bacterium was mixed in 5 mL of nutrient broth and kept overnight at 37 °C. The pathogenic bacteria *Staphylococcus aureus* MTTC 1809, *Bacillus subtilis* MTCC 430, *Salmonella enterica* MTCC 1253 and *Pseudomonas aeruginosa* MTCC 741 strains were gained from Egyptian Microbial Culture Collection, Microbiological Resource Center (The Cairo MIRCEN: Ain Shams University, Cairo, Egypt), cultured on a nutrient agar plate, and kept in the NA slants at 4 °C. Overnight cultures in the nutrient broth were used for the laboratory studies. The antibacterial activity of curcumin nanoparticles was estimated using the disc diffusion method [22], which was presented by the National Committee for Clinical Laboratory Standards (NCCLS). The zone of inhibition was measured after a day of incubation at 30 °C or 37 °C. Bactericidal effects of curcumin nanoparticles were detected using a modified version of the method shown by NCCLS. The diluted bacterial culture (0.1 mL) was extended on the sterile NA plate. Dried discs of 6 mm diameter of Whatman filter paper No. 1 that had been previously soaked in curcumin nanoparticles were placed on the seeded plates against Gram-negative and Gram-positive bacteria [23,24,25]. The estimation of the MIC was obtained through the determination of the turbidity of the bacterial growth after a day of incubation. The inhibited concentration was 99% of bacterial growth, which was considered as the MIC estimate [24,26]. According to the standard method, the MBC values of the particles were measured, and the MBC estimate was determined through sub-culturing the MIC dilutions onto sterile Muller-Hinton agar plates incubated at 37 °C for one day.

### 2.4. Experimental Design and Diets 

The study was carried out at the Poultry Research Farm, Department of Poultry, Faculty of Agriculture, Zagazig University, Egypt. At one week of age, we used 270 Japanese quails with an average body weight of 26.1 ± 0.08 g. Quail chicks were haphazardly distributed across six equal groups, each group consisting of 45 unsexed birds with five replications (nine birds each). Quails were reared in common type cage (90 × 40 × 40 cm) under the same conditions with 23 h light:1 h dark. Feed and water were opened throughout the experiment (five weeks). Birds received feeds in mash form according to their treatment. The dietary treatments were as follows: the 1st group was fed a basal diet without any supplementation (0 g/kg diet), whereas the 2nd, 3rd, 4th, 5th, and 6th groups were fed diets supplemented with 0.1, 0.2, 0.3, 0.4 and 0.5 g/kg of nano-curcumin, respectively. The basal diet was based on corn-soybean meal and contained 24% CP, 12.53 MJ/kg, 0.8 Ca, and 0.45 P, according to NRC [27]. 

### 2.5. Growth Performance and Carcass Measurements

All growth parameters [body weight (BW), body weight gain (BWG) feed intake (FI) and feed conversion ratio (FCR = g feed/g gain)] were measured at 1, 3 and 5 weeks of age. At 5-weeks-old, 24 quails were used for carcass examinations. All edible parts (gizzard, liver, heart, and carcass) were weighed and expressed as a % of the live BW before slaughter. 

### 2.6. Microbiological Analysis

We collected the samples (~10 g) from the cecum content (five samples per each treatment) and separately transported them to a 250 mL Erlenmeyer flask containing 90 mL of sterile peptone (0.1% peptone) saline solution (0.85% NaCl) and blended the mixture well. The total bacterial count (TBC), total yeasts and molds count (TYMC), *Enterococci*, lactic acid bacteria count, Coliform, *E. coli* and *Salmonella* were recorded according to [28,29].

### 2.7. Blood Chemistry 

After slaughter by sharp knife to complete bleeding, we collected the blood samples from 24 quails in sterilized tubes. We used the centrifuge (Janetzki, T32c, 5000 rpm, Wall-hausen, Germany) at 2000× *g* 15 min to separate the plasma. Using commercial kits from Biodiagnostic Company (Giza, Egypt), we determined the level of albumin (ALB), total protein (TP), globulin (GLOB), A/G ratio, and the activity of alanine transaminase (ALT), lactate dehydrogenase (LDH), aspartate transaminase (AST), urea, creatinine, total cholesterol (TC), triglycerides (TG), very low-density lipoprotein (VLDL), high-density lipoprotein (HDL), and low-density lipoprotein (LDL). The levels of immunological parameters (IgG) and M (IgM) as well as complement (C3) were determined using kits from Spectrum Company (Cairo, Egypt). For the antioxidant parameters, using commercial kits and a spectrophotometer (Shimadzu, Japan), the content of reduced glutathione (GSH) and malondialdehyde (MDA), and the activity of superoxide dismutase (SOD) were determined in quail plasma.

### 2.8. Statistics

All of the statistical analyses were carried out using the SAS software (SAS Institute Inc., Cary, NC, USA). Data regarding growth, carcass, blood chemistry and microbiology traits were analyzed with one-way ANOVA using the post-hoc Tukey’s test (*p* < 0.05).

## 3. Results 

The antibacterial activity of synthesized curcumin nanoparticles was tested against *Staphylococcus aureus* MTTC 1809, *Bacillus subtilis* MTCC 430, *Salmonella enterica* MTCC 1253 and *Pseudomonas aeruginosa* MTCC 741. The antibacterial activity against G+ and G- bacteria at different concentrations of curcumin nanoparticles was performed using the disc diffusion method. The bacterial growth culture at 24 h (0.1 mL) was spread aseptically onto nutrient agar plates. Discs (6 mm) which were impregnated with one of each curcumin nanoparticle concentration were dispensed with a sufficient separation from each other so as to avoid the overlapping of the inhibition zones. The diameters of the inhibition zone around each disc were estimated. They showed good antimicrobial activity against all the tested bacteria, though the effect of curcumin nanoparticles was found to be more pronounced with *Staphylococcus aureus* MTTC 1809 and *Bacillus subtilis* MTCC 430 (Table 1) compared to other bacteria. The data in Table 1 show the susceptibility of four bacterial strains to five concentrations of curcumin nanoparticles, namely 100, 200, 300, 400 and 500 µg/mL. The data confirmed that, with increasing concentrations of curcumin nanoparticles, halo diffusion increased regardless of the bacterial strains tested—giving a maximum diameter of 31 mm for *Staphylococcus aureus* MTTC 1809 when 500 µg/mL was used. From the obtained results, it was also observed that curcumin nanoparticles display more efficient as antibacterial activity compared to normal curcumin. The MIC values were 90, 100, 200 and 220 µg/mL, respectively, when *Staphylococcus aureus* MTTC 1809, *Bacillus subtilis* MTCC 430, *Salmonella enterica* MTCC 1253 and *Pseudomonas aeruginosa* MTCC 741 were used. The MBC rates were 180 µg/mL, 200 µg/mL, 400 µg/mL and 440 µg/mL, respectively, for the same pathological bacterial isolates mentioned earlier (Table 2).

### 3.1. Growth Performance and Carcass Yield 

Results for growth performance are shown in Table 3. It was found that the nano-curcumin levels significantly increased (*p* < 0.0001) body weight at 3 weeks and 5 weeks of age. It was reported that the diet enriched with nano-curcumin at levels of 0.2 or 0.4 g/kg resulted in the best body weight. Body weight gain during all periods (1–3, 3–5 and 1–5 weeks of age) was increased (*p* < 0.0001) in the groups treated with nano-curcumin levels, except the BWG of the group fed nano-curcumin (0.3 g/kg), which did not significantly differ from the control between 1 and 3 weeks of age. Between 1 and 3 weeks, the FI was significantly lower (*p* < 0.0001) with the supplementation of nano-curcumin (0.3 g/kg) than in all other groups, while the highest FI was with the birds fed on a diet containing nano-curcumin (0.2 g/kg). During the period of weeks 3–5, quails fed 0.1, 0.2, 0.3 and 0.4 g nano-curcumin-treated diets consumed less feed (*p* < 0.0001) than the others. Between 1 and 5 weeks, the FI was decreased (*p* < 0.0001) in the birds that received nano-curcumin (0.1, 0.3 and 0.4 g/kg) diets compared with that of the control and other groups. In all periods, the quails fed nano-curcumin had better FCR (*p* < 0.0001) than the control quails, except those quails fed nano-curcumin (0.5 g/kg) did not significantly differ from the control between 3 and 5 weeks of age. Generally, the best value in FCR was recorded for the 0.4 g nano-curcumin-treated group. As shown in Table 4, the carcass traits of Japanese quail were not affected by variation in nano-curcumin levels.

### 3.2. Blood Chemistry 

The effects of dietary nano-curcumin on the liver and kidney function of quail are shown in Table 5. The inclusion of nano-curcumin (0.2, 0.3 or 0.5 g/kg) increased serum TP (*p* = 0.0004) and globulin (*p* < 0.0001) compared to the control and other groups. The group fed nano-curcumin (0.3 g/kg) had the highest serum albumin level (*p* = 0.0078). The A/G ratio in the group fed 0.2, 0.3 and 0.5 g nano-curcumin/kg diet was lower than that in the control and other groups (*p* < 0.0001).

The AST activity in the serum decreased (*p* = 0.0116) with the addition of dietary nano-curcumin (0.1, 0.3 or 0.4 g/kg) to the feed. Furthermore, the serum ALT activity of the birds fed nano-curcumin (0.3 g/kg) was lower (*p* < 0.0001) than of those in the control and the other groups. The LDH values of the birds fed rations enriched with nano-curcumin (0.1, 0.2 or 0.4 g/kg) were lower (*p* = 0.0105) than of those in the other groups. There was no significant difference in the serum urea and creatinine values between birds supplemented with nano-curcumin at all levels and the control group.

The response of quails to dietary nano-curcumin levels on the lipid profile is presented in Table 6. The addition of various levels of nano-curcumin in the quail feed significantly decreased the TC and LDL in the serum (*p* < 0.0001) compared to the control. The highest values of HDL (*p* = 0.0308) were recorded with the group fed a diet containing nano-curcumin (0.4 g/kg). The TG and VLDL values were significantly decreased (*p* < 0.0001) with the addition of nano-curcumin (0.2, 0.4 and 0.5 g/kg) compared with the control and other groups, but the highest values were recorded for the 0.1 g/kg level of nano-curcumin.

The results of the antioxidant and immunity indices are presented in Table 7. Quails fed with nano-curcumin (0.2 g/kg) exhibited the highest SOD and GSH activities. However, MDA concentrations of serum were decreased by the addition of dietary nano-curcumin levels. In Table 7, serum IgG concentrations were increased by nano-curcumin (0.1, 0.2 and 0.5 g/kg) supplementation compared to those of the control. However, serum IgM concentrations were also increased by nano-curcumin (0.1, 0.2, 0.3 and 0.4 g/kg) supplementation compared to those of the control. The values of complement 3 were significantly augmented in the group fed diets containing nano-curcumin (0.2, 0.3, 0.4 and 0.5 g/kg).

### 3.3. Microbiological Aspects 

Table 8 presents the effect of nano-curcumin on the caecal microbiota of quail. The significant reduction in TBC, TYMC and *Enterobacter* in the caecal microbiota of quail was observed following the supplementation of nano-curcumin levels. The coliform count in the caecal microbiota of quail was significantly decreased in those groups fed a diet containing nano-curcumin (0.1, 0.4 and 0.5 g/kg) compared to in the other groups. Furthermore, the supplementation of nano-curcumin (0.2, 0.4 and 0.5 g/kg) led to a reduction in the caecal *E. coli* count compared to the control and other groups. Quails fed diets supplemented with nano-curcumin (0.2, 0.3 and 0.4 g/kg) exhibited higher lactic acid bacteria colonization than those in the control and other groups. The *Salmonella* counts in the caecal microbiota of quails were significantly decreased in all of the groups fed diets containing nano-curcumin. Finally, the best caecal microbiota were observed in the groups fed nano-curcumin at levels of 0.2 and 0.4 g/kg.

## 4. Discussion

Concerning the antimicrobial activity of curcumin nanoparticles against tested pathogenic bacteria, it was found [24] that when studying the effect of curcumin nanoparticles on four bacterial isolates, Gram-positive bacteria are more affected than Gram-negative bacteria. Furthermore, it was reported [24] that the antibacterial activity of nanocurcumin against *S. aureus*, *B. subtilis*, *E. coli*, and *P. aeruginosa* demonstrated a broad-spectrum inhibitory effect against all microorganisms. The MICs of nano-curcumin for *S. aureus*, *B. subtilis*, *E. coli*, and *P. aeruginosa* were 100, 75, 250, and 200 μg/mL, respectively.

The trend of using nano-curcumin in poultry feed has recently been discussed and may be a possible approach to enhance the physiological and productive performance, and the health status, of poultry. The enhancement of curcumin bioavailability using nanotechnology techniques can increase its absorption [30], in turn boosting the poultry performance and health. Nanocurcumin can be used as a safe and natural feed additive to increase nutritional value [16]. Supplemental nanocurcumin displayed a positive impact on BW and FCR, which is in accordance with previous studies [13,31], which validated the affirmative effect of curcumin on the growth performance of birds. As for the present findings, it was indicated [32] that the addition of 10 mg of nano-encapsulated curcumin/kg diet improved the FCR of quail. It was also illustrated [33] that the addition of nano-curcumin in the drinking water of broiler chicks improves the body growth and FCR. Curcumin, when used as a functional molecule, can act as a growth promoter in poultry, and as a strong natural antioxidant in the improvement of performance [34]. Additionally, using curcumin in the diet encourages the secretion of bile acids and stimulates the proteases lipase, amylase trypsin and chymotrypsin enzymes [35]. The favorable impacts of curcumin on the growth of broilers might be due to boosted secretions of these enzymes. The improvement in the growth of birds fed diets containing curcumin may be due to improvements in the intestinal morphology of the birds [13]. Furthermore, this positive effect of curcumin might be attributed to its well-reported antibacterial, antioxidant and anti-inflammatory effects [36]. Finally, nanocurcumin can be used in the poultry industry as a potential, promising feed additive. 

The results of the current study on the carcass yield of quail were in agreement with previous articles that studied the effects of turmeric or its extract on carcass traits, which were not affected. In Durrani et al. [31], higher breast and thigh weights, and a higher dressing percentage, were noticed in broilers fed a diet enriched with 5 g turmeric powder/kg compared with the control. A previous study [37] observed no improvement in the gizzard or liver following the application of *Curcuma longa* in the diet. Furthermore, it was stated [38] that the addition of turmeric rhizome extract (TRE) (100–300 mg/kg) had no significant influence on the dressing %. Moreover, it was found [39] that the gizzard and dressing % were not significantly influenced by dietary turmeric treatments.

The levels of blood ALT and AST reflect the health status of the liver. In the present study, these enzymes were significantly decreased following the addition of dietary nano-curcumin. Looking at previous research [14], it was clarified that supplementation of nanocurcumin (200 mg/kg) declined the serum AST enzyme level, and they attributed this reduction to the antioxidant properties of nano-curcumin. Furthermore, one study [40] reported that chickens fed a diet containing curcuma powder (5 g/kg) had the highest level of LDH, implying that curcuma might have a positive impact on liver enzymes. Moreover, it was highlighted [41] that a nano-curcumin level of 400 mg/kg diet displayed affirmative and consistent influences on the serum biochemical parameters. 

Serum TG, TC, HDL and LDL concentrations are viewed as diagnostic markers in lipid metabolism. The present results indicated that the addition of nano-curcumin levels in the quail feed significantly decreased the lipid profile. In agreement with previous studies [42,43] it was stated that curcumin reduced the serum LDL cholesterol and triglycerides levels and improved the liver function. Dietary curcumin lessened blood cholesterol levels and encouraged the digestion of fat [44,45]. Research [40] has revealed that the dietary supplementation of turmeric in broiler chickens significantly diminished LDL-cholesterol and augmented HDL-cholesterol, but did not affect triglyceride levels. Moreover, it was indicated [43,46] that curcumin caused a reduction in TC, perhaps due to the inhibition of enzyme hepatic 3-hydroxyl-3-methyglutaryl CoA-reductase (HMGCR) activity, which is responsible for the production of TC in the hepatic tissues [47]. Furthermore, curcumin may decline the activity of the enzymes that act as rate-limiting enzymes in lipogenesis, such as acetyl-CoA carboxylase (the rate-limiting enzyme in fatty acids synthesis) [48].

Antioxidant ability is the key to the health and growth of poultry. Curcumin, the major antioxidative molecule of curcuma longa, is a powerful damper of oxygen species [49]. The results of the current study show that the groups fed with nano-curcumin exhibited high SOD and GSH activities. As shown by the present findings, nano-curcumin possesses a better antioxidant and biological activity than curcumin [50]. The antioxidant ability against peroxyl radicals was augmented in the nanocurcumin-supplemented group compared to the control [32]. The curcumin can alleviate the oxidative stress by modifying the hepatic nuclear transcription factors and decreasing lipid peroxidation in the muscle and serum of quail [51]. Curcumin helps to maintain the antioxidant status of the cells through suppressing oxidative enzymes, scavenging free radicals and prompting de novo glutathione synthesis [52]. Zhai et al. [15] illustrated that curcumin compounds could reduce the oxidative injury and disruption of lipid metabolism through modifying the cecum microbiota of ducks. The dietary supplementation of turmeric rhizome extract augmented the enzymatic activities of SOD and GSH-PX, and reduced the malondialdehyde concentration [38]. As a result, dietary curcumin reduces the production of reactive free radicals, leading to an increase in the antioxidant metabolites concentration in the poultry body. The inclusion of curcumin in the diet decreased the malondialdehyde concentration and boosted the activities of CAT, T-AOC, SOD, and GSH-Px compared to the control group [53]. Thus, curcumin can alleviate the negative impact of any stressful environmental condition.

Curcumin has been found to have numerous pharmacological activities, including antimicrobial, anti-inflammatory, antifungal, antiviral and antioxidant activities [54]. The highest values of immunoglobulins in the present study were obtained from birds fed a diet containing nano-curcumin. Similar results have been observed in other studies, which pointed out that a diet with a nano-curcumin level of 400 mg/kg displayed the best immune response compared to the control group [41]. Emadi et al. [55] showed that the immunologlobulins (IgA, IgM and IgG) of chickens were significantly increased by dietary turmeric. Furthermore, the turmeric plant has been proven to be a powerful immunomodulatory factor that can improve the activation of B and T cells, neutrophils and macrophages cells [56]. Thus, it can be said that nano-curcumin can be an appropriate alternative to synthetic antioxidants, perhaps due to the improvement of the antioxidant metabolites of birds, which may boost the bird’s immunity.

The present study found that the supplementation of nano-curcumin in quail diets reduced harmful bacteria and boosted useful bacteria. Curcumin, the main bioactive component of turmeric, was found to possess antibacterial activities [57]. Curcumin could modify the gut microbial balance, improving the intestinal integrity [58]. El-Rayes et al. [39] illustrated that the dietary supplementation of turmeric as source of curcumin led to an increase in counts of lactic acid bacteria and a reduction in pathogenic bacteria (*S. aureus*, *E. coli* and total coliform bacteria) compared to in the control group. Gupta et al. [59] described that the extracts of *C. longa* inhibited the growth of pathogenic bacteria. Curcumin had an inhibitory effect against many pathogenic bacteria and decreased the population of harmful gut bacteria [60].

## 5. Conclusions

The findings of our study demonstrated the positive effects of dietary nano-curcumin supplementation on the growth, lipid profile, blood constituents, immunity and antioxidant indices of quails. The best values in feed efficiency were achieved when quails were fed with 0.4 g nano-curcumin in their diets. Furthermore, the best values in immune response and antioxidant indices were observed in the 0.2 g nano-curcumin-treated group.

## Figures and Tables

**Table 1:** Zone of inhibition produced by curcumin and curcumin nanoparticles.

Items	Curcumin (100 μg/mL)	Curcumin nanoparticles (μg/mL)					SEM	*p* Value
		100	200	300	400	500		
*Staphylococcus aureus* MTTC 1809	13.33 ^e^	19.33 ^d^	21.67 ^d^	25.67 ^c^	28.33 ^b^	31.00 ^a^	0.831	<0.0001
*Bacillus subtilis* MTCC 430	14.67 ^e^	19.00 ^d^	20.67 ^d^	23.33 ^c^	26.33 ^b^	28.67 ^a^	0.612	<0.0001
*Salmonella enterica* MTCC 1253	12.33 ^e^	15.67 ^d^	18.67 ^c^	21.33 ^b^	23.67 ^ab^	26.00 ^a^	0.793	<0.0001
*Pseudomonas aeruginosa* MTCC 741	11.33 ^d^	13.67 ^d^	17.33 ^c^	19.67 ^bc^	21.67 ^ab^	23.33 ^a^	0.882	<0.0001

^a–e^: Means in the same row with no superscript letters after them or with a common superscript letter following them are not significantly different (*p* < 0.05).

**Table 2:** The MIC (Minimum Inhibitory Concentration) and MBC (Minimum Bactericidal Concentration) of the curcumin nanoparticles.

Pathogenic Bacteria	Curcumin Nanoparticles	
	MIC µg/mL	MBC µg/mL
*Staphylococcus aureus* MTTC 1809	90	180
*Bacillus subtilis* MTCC 430	100	200
*Salmonella enterica* MTCC 1253	200	400
*Pseudomonas aeruginosa* MTCC 741	220	440

**Table 3:** Growth performance of Japanese quail as affected by dietary nanocurcumin.

Items	Nano-curcumin Level (g/kg Diet)						SEM	*p* Value
	0	0.1	0.2	0.3	0.4	0.5		
**Body Weight (g)**								
1 week	26.00	26.15	26.10	26.22	26.15	26.17	0.075	0.5406
3 weeks	90.33 ^c^	96.84 ^b^	100.23 ^ab^	90.87 ^c^	102.20 ^a^	98.45 ^ab^	1.157	0.0001
5 weeks	173.08 ^d^	181.63 ^bc^	189.14 ^a^	178.13 ^c^	191.19 ^a^	182.68 ^b^	1.091	<0.0001
**Body Weight Gain (g/day)**								
1–3 weeks	4.59 ^c^	5.05 ^b^	5.29 ^ab^	4.62 ^c^	5.43 ^a^	5.16 ^ab^	0.083	0.0002
3–5 weeks	5.91 ^d^	6.06 ^c^	6.35 ^a^	6.23 ^b^	6.36 ^a^	6.02 ^c^	0.026	<0.0001
1–5 weeks	5.25 ^d^	5.55 ^bc^	5.82 ^a^	5.43 ^c^	5.89 ^a^	5.59 ^b^	0.039	<0.0001
**Feed Intake (g/day)**								
1–3 weeks	14.21 ^bc^	13.91 ^c^	15.42 ^a^	13.22 ^d^	13.76 ^cd^	14.54 ^b^	0.152	<0.0001
3–5 weeks	22.96 ^a^	21.65 ^b^	22.04 ^b^	19.74 ^c^	19.97 ^c^	23.10 ^a^	0.227	<0.0001
1–5 weeks	18.59 ^a^	17.78 ^b^	18.73 ^a^	16.48 ^c^	16.86 ^c^	18.82 ^a^	0.153	<0.0001
**Feed Conversion Ratio (g/g)**								
1–3 weeks	3.09 ^a^	2.75 ^d^	2.91 ^b^	2.86 ^bc^	2.53 ^e^	2.82 ^cd^	0.020	<0.0001
3–5 weeks	3.88 ^a^	3.58 ^b^	3.47 ^b^	3.17 ^c^	3.14 ^c^	3.84 ^a^	0.046	<0.0001
1–5 weeks	3.54 ^a^	3.20 ^c^	3.22 ^c^	3.04 ^d^	2.86 ^e^	3.37 ^b^	0.021	<0.0001

^a–d^: Means in the same row with no superscript letters after them or with a common superscript letter following them are not significantly different (*p* < 0.05).

**Table 4:** Carcass traits and relative organs of growing Japanese quail as affected by dietary nano-curcumin.

Items	Nano-curcumin Level (g/kg Diet)						SEM	*p* Value
	0	0.1	0.2	0.3	0.4	0.5		
Carcass %	76.83	76.28	78.21	76.53	75.52	75.45	1.561	0.8558
Liver %	2.23	2.48	2.12	2.54	2.73	2.74	0.180	0.2600
Gizzard %	2.63	2.52	2.74	2.64	2.44	2.37	0.204	0.8648
Heart %	0.98	1.17	1.12	1.08	1.05	0.94	0.100	0.6537
Giblets %	5.85	6.17	5.98	6.27	6.23	6.04	0.276	0.9506
Dressing %	82.67	82.45	84.19	82.8	81.75	81.5	1.705	0.9170

**Table 5:** Liver and kidney function of growing Japanese quail as affected by dietary nano-curcumin.

Items	Nano-curcumin Level (g/kg Diet)						SEM	*p* Value
	0	0.1	0.2	0.3	0.4	0.5		
TP ^†^ (g/dL)	3.13 ^b^	3.07 ^b^	3.75 ^a^	3.70 ^a^	3.31 ^b^	3.61 ^a^	0.074	0.0004
ALB ^‡^ (g/dL)	1.24 ^bc^	1.23 ^bc^	1.14 ^c^	1.36 ^a^	1.31 ^ab^	1.24 ^bc^	0.028	0.0078
GLOB ^§^ (g/dL)	1.89 ^c^	1.84 ^c^	2.62 ^a^	2.35 ^b^	2.00 ^c^	2.38 ^b^	0.049	<0.0001
A/G ^¶^ (%)	0.65 ^a^	0.67 ^a^	0.44 ^d^	0.58 ^b^	0.66 ^a^	0.52 ^c^	0.015	<0.0001
AST ^††^ (IU/L)	181.20 ^a^	168.30 ^b^	169.87 ^ab^	164.20 ^b^	161.15 ^b^	180.70 ^a^	3.107	0.0116
ALT ^‡‡^ (IU/L)	14.64 ^b^	13.90 ^b^	14.50 ^b^	9.57 ^c^	12.85 ^b^	16.63 ^a^	0.532	<0.0001
LDH * (IU/L)	196.40 ^a^	157.85 ^b^	165.60 ^b^	190.07 ^a^	167.80 ^b^	206.70 ^a^	5.159	0.0002
Creatinine (mg/dL)	0.34	0.31	0.33	0.34	0.36	0.37	0.017	0.3588
Urea (mg/dL)	6.82	6.55	6.84	7.22	7.07	7.09	0.128	0.0970

^a–d^: Means in the same row with no superscript letters after them or with a common superscript letter following them are not significantly different (*p* < 0.05). ^†^ TP: total protein; ^‡^ Alb: albumin ^§^ GLOB: globulin; ^¶^ A/G: albumin/globulin ratio; ^††^ AST: aspartate aminotransferase and ^‡‡^ ALT: alanine aminotransferase. * LDH: lactate dehydrogenase.

**Table 6:** Lipid profile of growing Japanese quail as affected by dietary nano-curcumin.

Items	Nano-curcumin Level (g/kg Diet)						SEM	*p* Value
	0	0.1	0.2	0.3	0.4	0.5		
TC ^†^ (mg/dL)	259.15 ^a^	176.75 ^cd^	195.45 ^b^	182.95 ^c^	172.00 ^cd^	167.17 ^d^	3.155	<0.0001
TG ^‡^ (mg/dL)	218.15 ^b^	296.98 ^a^	137.21 ^d^	208.48 ^b^	186.25 ^c^	169.83 ^c^	5.167	<0.0001
HDL ^§^ (mg/dL)	44.56 ^b^	49.01 ^ab^	51.15 ^ab^	43.64 ^b^	56.51 ^a^	51.87 ^ab^	2.188	0.0308
LDL ^¶^ (mg/dL)	170.97 ^a^	68.35 ^d^	116.86 ^b^	97.61 ^c^	78.24 ^d^	81.33 ^d^	3.960	<0.0001
VLDL^¶¶^ (mg/dL)	43.63 ^b^	59.40 ^a^	27.44 ^d^	41.70 ^b^	37.25 ^c^	33.97 ^c^	1.033	<0.0001

^a–d^: Means in the same row with no superscript letters after them or with a common superscript letter following them are not significantly different (*p* < 0.05). ^†^ TC: total cholesterol; ^‡^ TG: triglycerides; ^§^ HDL: high density lipoprotein; ^¶^ LDL: low density lipoprotein; ^¶¶^ LDL: very low density lipoprotein.

**Table 7:** Antioxidant and immunological indices of growing Japanese quail as affected by dietary nano-curcumin.

Items	Nano-curcumin Level (g/kg Diet)						SEM	*p* Value
	0	0.1	0.2	0.3	0.4	0.5		
SOD ^†^ (U/mL)	0.23 ^b^	0.22 ^b^	0.37 ^a^	0.25 ^b^	0.24 ^b^	0.24 ^b^	0.010	<0.0001
MDA ^‡^ (nmol/mL)	0.17 ^a^	0.19 ^a^	0.10 ^c^	0.11 ^c^	0.14 ^b^	0.12 ^c^	0.006	<0.0001
GSH ^¶^ (ng/mL)	0.25 ^b^	0.23 ^b^	0.39 ^a^	0.26 ^b^	0.25 ^b^	0.28 ^b^	0.012	0.0002
IgG ^§§^ (mg/dl)	1.00 ^c^	1.30 ^ab^	1.45 ^a^	1.15 b ^c^	1.05 ^c^	1.32 a^b^	0.061	0.0047
IgM ^§§^ (mg/dl)	0.65 ^b^	1.01 ^a^	1.09 ^a^	1.01 ^a^	1.08 ^a^	0.54 ^b^	0.059	0.0002
C3 ^‡‡^ (mg/dl)	128.00 ^e^	138.50 ^e^	177.50 ^d^	199.50 ^c^	311.50 ^b^	405.00 ^a^	3.657	<0.0001

^a–d^: Means in the same row with no superscript letters after them or with a common superscript letter following them are not significantly different (*p* < 0.05). ^†^ SOD: superoxide dismutase; ^‡^ MDA: malondialdehyde; ^§^ TAC: total antioxidant capacity; ^¶^ GSH: reduced glutathione; ^††^ GPX: glutathione peroxidase; ^‡‡^ C3: complement 3; ^§§^ IgG: immunoglobulin G.

**Table 8:** Caecal microbiota of growing Japanese quail as affected by dietary nano-curcumin.

Items	Nano-curcumin Level (g/kg Diet)						SEM	*p* Value
	0	0.1	0.2	0.3	0.4	0.5		
**Microbiological Count (Log CFU/g)**								
TBC	6.07 ^a^	5.13 ^d^	6.00 ^b^	5.30 ^c^	6.03 ^b^	5.14 ^d^	0.009	<0.0001
TYMC	5.88 ^a^	4.82 ^d^	5.74 ^b^	5.78 ^b^	5.12 ^c^	4.88 ^d^	0.025	<0.0001
*Coliform*	5.98 ^a^	5.01 ^c^	5.94 ^a^	5.94 ^a^	5.23 ^b^	5.05 ^c^	0.019	<0.0001
*E. coli*	5.94 ^a^	5.86 ^a^	4.96 ^c^	5.90 ^a^	5.20 ^b^	5.01 ^c^	0.025	<0.0001
Lactic acid bacteria	5.24 ^c^	5.04 ^d^	5.91 ^b^	5.97 ^b^	6.03 ^a^	5.08 ^d^	0.016	<0.0001
*Enterobacter* spp.	5.92 ^a^	4.91 ^d^	5.82 ^b^	5.83 ^b^	5.16 ^c^	4.94 ^d^	0.023	<0.0001
*Salmonella* spp.	6.46 ^a^	3.60 ^b^	2.13 ^c^	1.36 ^d^	0.98 ^e^	0.26 ^f^	0.067	<0.0001

^a–d^: Means in the same row with no superscript letters after them or with a common superscript letter following them are not significantly different (*p* < 0.05); TBC: Total bacterial count; TYMC: total yeast and molds count.
